# Incidents of Practice

**Published:** 1903-04

**Authors:** 


### Incidents of Practice.

Treatment for Putrescent Pulp-Canals.—In connection with the treatment of canals, Dr. F. W. Stephan writes as follows: "In the treatment of abscesses and of root-canals generally I have found the following mixture most serviceable. Take of oil of cloves and carbolic acid crystals, equal parts. Melt the carbolic acid crystals by heat and add the oil of cloves. This mixture is easily prepared and possesses in marked degree the desirable qualities of the essential oil,—carbolic acid mixture. If used with reasonable care it will not discolor the teeth in which it is placed.—*Items of Interest.*

Beta-Eucaine.—In a brochure on "Local Anæsthesia and its Application in Dentistry" (Leipsic, Arthur Felix, 1902), Dr. Herman Thiesing, Royal Court Dentist, discusses the twelve anæsthetics which have been used in stomatology. He finds that acoine, cocaine, alpha-eucaine, holocaine, orthoform, and orthoform-new are less suited because of their greater toxicity; nirvanin, anesin, etc., because of insufficient anæsthetic action and other disadvantages; and that beta-eucaine should be employed. Dr. Thiesing has also used Wilson's anæsthetic, but has entirely ceased to employ it, having become convinced that better results are obtained with a one per cent. beta-eucaine solution; moreover, Wilson's anæsthetic is fifteen times as expensive as such a solution.

Beta-eucaine possesses greater anæsthetic power than tropacocaine, while the duration and intensity of the anæsthesia, and the area affected by it, are equal to that of cocaine. Besides being three and three-fourths less toxic than cocaine, beta-eucaine possesses the important advantage that its solutions can be sterilized with boiling. The author has never observed œdemas or subsequent pains from the use of boiled beta-eucaine solutions, though sometimes, just as with tropacocaine, bleedings followed its employment, which, however, have always stopped promptly without aid.

Insensibility of the mucous membrane may be easily produced by the external application of a beta-eucaine solution. The injection of a one per cent. solution (to which eight-tenths of one per cent. sodium chloride have been added) at body temperature is absolutely painless and effects a thorough anæsthesia lasting twenty-five or thirty minutes.

From his investigations Dr. Thiesing draws the conclusion that in dentistry only beta-eucaine and tropacocaine may be considered, and occasionally, if very dilute solutions are to be used, or for application to the unbroken skin, also acoine and cocaine.

He himself prefers beta-eucaine because it is less toxic than tropacocaine and has considerably more anæsthetic power. According to the investigations made by various physicians, a one per cent. beta-eucaine solution produces about the same effect as a four per cent. tropacocaine solution. The possibility of intoxication is therefore much greater with tropacocaine.

Dr. Thiesing considers it advisable to employ beta-eucaine solutions of four different strengths for various purposes. He indicates *2.5 per cent. beta-eucaine solutions* for opening abscesses, excising small tumors, extracting loosened (not inflamed) teeth and roots (deciduous teeth) and the four lower incisors, and anæsthetizing the inferior alveolar nerve on the lingula and on the foramen mentals; *one per cent. beta-eucaine solutions* for extracting the upper incisors, bicuspids, and molars, straightening teeth by operation, drilling the alveola, and removing necrotic root apexes; *two per cent. beta-eucaine solutions* for extracting the lower molars and bicuspids and the four canines, excavating sensitive dentines, and extracting the pup; and *three per cent. beta-eucaine solutions* for extracting all teeth and roots in acute peridontitis.—*Items of Interest.*

---

Carbolic Acid as a Coagulator.—At the American Dental Society of Europe meeting Dr. E. Lawley York reaffirmed that carbolic acid in the pulp-canal of a tooth penetrated the dentine completely, but not the cementum, hence its safety. In cases of freshly removed pulps a sufficient quantity of the material dissolved its own coagulum, and in putrescence the decomposing process has destroyed all albuminous contents of the tooth.—H. L. W., *Dental Review,* January, 1903.

Gutta-percha for setting Inlays.—Dr. Hofheinz, of Rochester, N. Y., suggests the use of gutta-percha for setting inlays in those cases where from some untoward condition of the oral fluids cement is rapidly disintegrated. He uses for this purpose a solution of gutta-percha in nine parts chloroform and one part eucalyptus oil, the latter being added to retard the evaporation of the chloroform. After the inlay is made, the inner surface is roughened in the usual way, coated with a light-colored ether varnish, and laid aside until the ether of the varnish has evaporated. In the mean while the cavity in the tooth is deepened, slightly undercut, and the undercuts filled with cement. The inlay is carefully held with a pair of tweezers while the edges are moistened with the gutta-percha solution; it is then pressed into place while the cement is still plastic. Much care is needed to accurately gauge the amount of cement placed in the cavity, so that, while there is enough, there is no surplus to dislodge the gutta-percha on the edges of the inlay. By thus combining cement and gutta-percha in setting the inlay, the doctor hopes to secure the firmness and strong adhesion of the cement, and at the same time protect the cement from disintegration by interposing the gutta-percha, which alone comes in contact with the fluids of the mouth.—*Dental Cosmos,* January, 1903, page 31.

---

A New Obtundent.—Dr. Hofheinz, of Rochester, N. Y., suggests the use of zinc chloride in an alcoholic and chloroform solution instead of an aqueous one, for the treatment of hypersensitive dentine. The pain is greatly lessened, and it acts more rapidly owing to the desiccating and obtunding effect of the chloroform and alcohol.—*Dental Cosmos,* January, 1903, page 33.

---

Deaths from swallowing False Teeth.—In the *Dental Record* (London), February, 1903, page 97, four cases of death from swallowing false teeth are noted, all occurring within a few weeks, a very unusual form of epidemic. In three cases the accident happened during sleep; in the fourth it followed a hearty laugh, death resulting quickly from choking.

In the three cases first mentioned the victims were in the habit of wearing their plates at night. In the first, after other means had failed to give relief, the plate, carrying three teeth, was removed by an operation, from the after-effects of which and heart weakness the patient succumbed.

In the second, owing to its position, it was three days before the medical men in charge succeeded in removing the plate. In the mean while septic inflammation had set in, and this condition caused death.

In the third, a man, aged sixty-eight years, became suddenly ill, but refused to see a doctor. A few days later he was found dead in bed. A post-mortem examination revealed some false teeth firmly fixed in the dead man's throat. The sharp edge of the plate had ruptured the small blood-vessels, and this had brought about death.

These cases show the importance of removing artificial dentures before going to bed. There is the further consideration that it is only by such removal that thorough cleansing and disinfection of the plates can be secured.

---

Dovetail Porcelain Inlays.—In the *Dental Record* (London), February, 1903, page 103, a somewhat novel method of making and securing porcelain inlays, introduced by Mr. F. R. Howard, is described and illustrated. It is a modification of a method for inserting porcelain inlays in the approximal surfaces of anterior teeth introduced some time ago, in which, by means of suitable revolving tools, the cavity is made to assume the form of a cylindrical tapered hole, horizontally across the approximal surface of the tooth, about two-thirds of its circumference being formed within tooth-tissue. Into this a portion of a porcelain rod, accurately ground to fit the opening made by the tool, is cemented, and shaped to conform to the contour of the tooth after the cement has become hard. Mr. Howard, by means of fissure burs and a special file transforms the approximal cavity into one having three absolutely flat walls arranged to form a slight dovetail. He thus avoids making the attenuated frail edges of tooth-tissue which formed a portion of the cavity walls in the former system, and secures strong cavity margins. The opening is made to receive a portion of a porcelain rod tapering in its length from about onefourth to one-eighth of an inch, nearly oblong in section, one side being slightly wider than the other, so as to interlock or dovetail with the cavity formed in the tooth. These porcelain rods are prepared and furnished by the manufacturer accurately ground on three sides. Mr. Howard claims for his method that the repair of the tooth is almost invisible; that nearly all the cavity edge is exposed to the cleansing action of the lips and tongue; that the inlay is self-supporting, and that geometrical exactness is possible. Theoretically, it has much to commend it; it is doubtful, however, if in practice it will fully meet expectations.

